# Identification of Arginine Phosphorylation in Mycolicibacterium smegmatis

**DOI:** 10.1128/spectrum.02042-22

**Published:** 2022-10-10

**Authors:** Emmanuel C. Ogbonna, Henry R. Anderson, Karl R. Schmitz

**Affiliations:** a Department of Biological Sciences, University of Delaware, Newark, Delaware, USA; b Department of Chemistry & Biochemistry, University of Delaware, Newark, Delaware, USA; Penn State University

**Keywords:** *Mycobacterium tuberculosis*, mass spectrometry, phosphoarginine, phosphoproteomics, proteolysis, tuberculosis

## Abstract

Tuberculosis is a leading cause of worldwide infectious mortality. The prevalence of multidrug-resistant Mycobacterium tuberculosis infections drives an urgent need to exploit new drug targets. One such target is the ATP-dependent protease ClpC1P1P2, which is strictly essential for viability. However, few proteolytic substrates of mycobacterial ClpC1P1P2 have been identified to date. Recent studies in Bacillus subtilis have shown that the orthologous ClpCP protease recognizes proteolytic substrates bearing posttranslational arginine phosphorylation. While several lines of evidence suggest that ClpC1P1P2 is similarly capable of recognizing phosphoarginine-bearing proteins, the existence of phosphoarginine modifications in mycobacteria has remained in question. Here, we confirm the presence of posttranslational phosphoarginine modifications in Mycolicibacterium smegmatis, a nonpathogenic surrogate of M. tuberculosis. Using a phosphopeptide enrichment workflow coupled with shotgun phosphoproteomics, we identified arginine phosphosites on several functionally diverse targets within the M. smegmatis proteome. Interestingly, phosphoarginine modifications are not upregulated by heat stress, suggesting divergent roles in mycobacteria and *Bacillus*. Our findings provide new evidence supporting the existence of phosphoarginine-mediated proteolysis by ClpC1P1P2 in mycobacteria and other actinobacterial species.

**IMPORTANCE** Mycobacteria that cause tuberculosis infections employ proteolytic pathways that modulate cellular behavior by destroying specific proteins in a highly regulated manner. Some proteolytic enzymes have emerged as novel antibacterial targets against drug-resistant tuberculosis infections. However, we have only a limited understanding of how these enzymes function in the cell and how they select proteins for destruction. Some proteolytic enzymes are capable of recognizing proteins that carry an unusual chemical modification, arginine phosphorylation. Here, we confirm the existence of arginine phosphorylation in mycobacterial proteins. Our work expands our understanding of a promising drug target in an important global pathogen.

## INTRODUCTION

Tuberculosis is a leading cause of worldwide infectious mortality, ranking above HIV/AIDS and Ebola, and is one of the top 10 leading causes of death overall (1). Advances in diagnosis, vaccinations, and therapeutics have reduced tuberculosis morbidity and mortality. However, the prevalence of multidrug resistance in the causative bacterium, Mycobacterium tuberculosis, remains high (1). These statistics underscore the urgent need to discover new drugs and exploit new molecular targets. One promising target is the mycobacterial Clp protease. Several studies show that Clp protease components are strictly essential for M. tuberculosis viability (2–6) and are viable targets for anti-M. tuberculosis therapeutics (7–12).

Clp proteases mechanically unfold and destroy native cytosolic proteins (13, 14). These large enzymatic complexes consist of a core peptidase and an associated hexameric ATP-dependent unfoldase. The mycobacterial Clp peptidase, ClpP1P2, is a heteromer composed of distinct ClpP1 and ClpP2 rings that stack face to face to create a barrel-shaped tetradecamer (15–19). ClpC1 is one of two mycobacterial unfoldases (the other is ClpX) that can assemble with ClpP1P2 to form a functional protease. ClpC1 is an 848-residue protein with a globular N-terminal domain (NTD) and two AAA+ (ATPases associated with various cellular activities) modules. Ring-shaped hexamers of ClpC1 dock coaxially on the surface of the ClpP2 face of the peptidase (20, 21). Protein substrates destined for destruction by ClpC1P1P2 must first be recognized by ClpC1. The unfoldase therefore plays a critical regulatory role in proteolysis.

Clp proteases have become major targets of novel drug development against M. tuberculosis and other pathogenic bacteria. Several classes of antimicrobials specifically target the Clp peptidase, including dysregulators (e.g., acyldepsipeptides [22, 23]), activators (e.g., sclerotiamide [24]), and catalytic inhibitors (e.g., β-lactones and boronate compounds [7, 25]). Importantly, multiple cyclic peptides have been discovered that exhibit anti-M. tuberculosis activity through dysregulation of ClpC1, including cyclomarin A, lassomycin, ecumicin, rufomycin, and metamarin (8, 9, 26–28). These compounds bind to the ClpC1 NTD and disrupt proteolysis by uncoupling unfoldase activity from proteolysis or by causing uncontrolled degradation of cellular proteins. The antimicrobial activity of ClpC1-targeting compounds underscores the importance of ClpC1P1P2 in mycobacteria. However, the specific proteolytic functions responsible for its essentiality remain obscure, and only a few protein substrates have been identified (29–32).

An expanded understanding of the physiological roles played by ClpC1P1P2 would bolster efforts to develop antimicrobial compounds. In other bacteria, Clp proteases participate in cellular processes ranging from targeted pathway regulation to cell-wide protein quality control (33, 34). Multiple mechanisms of substrate recognition have been described, including direct interactions with the unfoldase (30, 31) and indirect recognition with the aid of adaptors (32, 35). Recent studies with Bacillus subtilis and related *Firmicutes* have demonstrated that posttranslational arginine phosphorylation marks some proteins for destruction by ClpCP (34, 36, 37). Dual phosphoarginine (pArg) binding sites on the B. subtilis ClpC NTD allow ClpCP to recognize phosphoarginylated proteins as proteolytic substrates (34). Phosphoarginine-mediated proteolysis in these bacteria is upregulated during stress through activation of the arginine kinase McsB (38). Phosphoproteomic studies have uncovered widespread arginine phosphorylation during heat stress, implicating ClpCP in turnover of misfolded proteins (34, 36, 39). Additionally, McsB regulates the global stress response through targeted phosphorylation of the negative transcriptional regulators CtsR and HrcA (36, 38, 40). Proteolysis of these targets by ClpCP allows transcriptional activation of stress response genes (38). Interestingly, ClpC and ClpP are themselves targets of McsB, and specific pArg sites on ClpC are required for its activation by McsB (36, 41). These studies underline the role of arginine phosphorylation in *Firmicutes* as both a degradation signal and a regulatory mechanism.

Several lines of evidence suggest that an analogous pArg-mediated proteolytic pathway exists in mycobacteria. Sequence and structural data reveal overall homology between the NTDs of M. tuberculosis ClpC1 and B. subtilis ClpC, as well as strong conservation of the residues surrounding the pArg-binding sites (34, 42). Moreover, *in vitro* experiments confirm that the M. tuberculosis ClpC1 NTD does indeed interact with both free pArg and with arginine-phosphorylated model substrates (42). However, the existence of pArg-mediated proteolysis in mycobacteria remains in question, as no mycobacterial McsB homologs are known and phosphoarginine modifications have not yet been described for these bacteria.

Here, we confirm the existence of posttranslational phosphoarginine modifications in Mycolicibacterium smegmatis, a nonpathogenic surrogate of M. tuberculosis. Using a phosphopeptide enrichment workflow coupled to shotgun phosphoproteomics, we identified arginine phosphosites on several protein targets within the M. smegmatis proteome. Our findings suggest that these modifications are widespread among actinobacterial species.

## RESULTS

### Phosphoarginine binding sites in the ClpC1 NTD are conserved across *Actinobacteria*.

**FIG 1:**
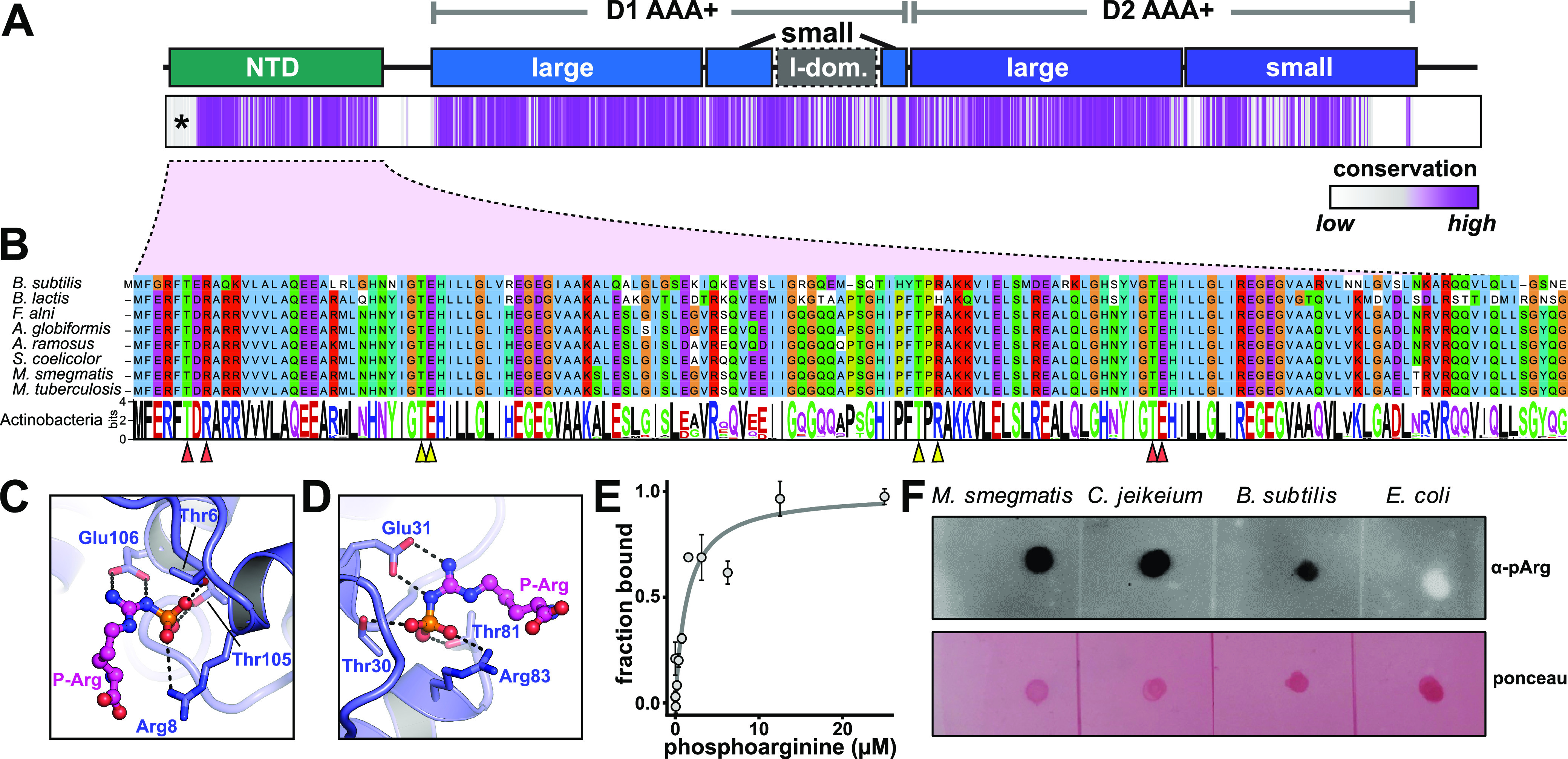
Indirect evidence for phosphoarginine in actinobacteria. (A) The domain organization of ClpC1 is shown above a plot of amino acid conservation among actinobacterial ClpC1 orthologs, where conservation at each alignment position is plotted as a purple vertical strip. The apparent poor conservation at the beginning of the NTD (marked by an asterisk) likely reflects misannotation of the start site in some entries. (B) The sequence of the Bacillus subtilis ClpC NTD is shown above equivalent regions from several actinobacterial ClpC orthologs. The sequence logo below shows amino acid conservation in actinobacterial ClpC1 orthologs. Arrows mark positions reported to be important for phosphoarginine binding to site 1 (orange) or site 2 (yellow) (34). Phosphoarginines from the crystal structure of the B. subtilis ClpC NTD (PDB code 5HBN) were modeled on the M. tuberculosis ClpC1 NTD (PDB code 6PBQ) putative phosphoarginine-binding sites 1 (C) and 2 (D) (47). (E) Binding of phosphoarginine to M. smegmatis ClpC1^NTD^ was measured by microscale thermophoresis. Data were fit to a noncooperative binding model (gray curve), yielding a *K_D_* (equilibrium dissociation constant) of 1.4 ± 0.6 μM. Values are averages of three technical replicates ± 1 SD. (F) Dot blots of *Mycolicibacterium smegmatis*, Corynebacterium jeikeium, Bacillus subtilis, and Escherichia coli cell lysates, probed with anti-phosphoarginine antibody (48) or stained with Ponceau S.

ClpC1 is an essential enzyme in M. tuberculosis and M. smegmatis (2, 6), yet few specific cellular roles or proteolytic substrates of the ClpC1P1P2 protease are known. To gain insight into its cellular function, we began by examining ClpC1 sequence conservation across the phylum *Actinobacteria* by constructing an alignment of 1,195 actinobacterial orthologs (Fig. 1A). Like most other type-II Clp unfoldases, ClpC1 possesses an ~150-amino-acid (aa) N-terminal domain (NTD), which likely participates in regulation and substrate recognition, but lacks mechanical function or a direct role in catalysis (35, 43). Surprisingly, the NTD is as well conserved as or better conserved than the D1 and D2 AAA+ ATPase rings, suggesting that the NTD has important and conserved functions across *Actinobacteria*. This may help explain why naturally occurring antibiotics that target and dysregulate ClpC1 have evolved to specifically bind the NTD (8, 9, 26, 44–46).

In Bacillus subtilis and other *Firmicutes*, ClpCP recognizes phosphorylated arginine residues as degradation signals via twin binding sites on opposite ends of the ClpC NTD (34). Examination of actinobacterial ClpC1 sequences revealed that the residues known to be important for phosphoarginine binding in B. subtilis are conserved across actinobacterial orthologs (Fig. 1B). We found that we could readily model bound phosphoarginines in an existing structure of the M. tuberculosis ClpC1 NTD (47), with minor side chain rearrangements, based on the observed mode of binding to the B. subtilis ClpC NTD (34) (Fig. 1C and D). Finally, we directly assessed binding of pArg to purified M. smegmatis ClpC1^NTD^ by microscale thermophoresis (Fig. 1E). Phosphoarginine bound with an affinity of 1.4 μM, similar to the ~5 μM affinity previously reported for the M. tuberculosis ClpC1 NTD (42). The ability of the ClpC1^NTD^ to bind pArg and the conservation of pArg binding modules across *Actinobacteria* provide strong indirect evidence for the existence of this posttranslational modification in this phylum.

### Identification of phosphoarginine modifications in *Mycolicibacterium smegmatis*.

To test for the physiological existence of arginine phosphorylation, we probed dot blots of several bacterial lysates with a phosphoarginine-specific antibody (Fig. 1F) (48). In line with prior studies, signal was detected in B. subtilis lysate (48) but not in lysate from Escherichia coli, which lacks arginine phosphorylation (49, 50). We also observed a positive immunoblot reaction in lysates from M. smegmatis and a second actinobacterium, Corynebacterium jeikeium. These observations suggest that pArg modifications occur at least within the suborder *Corynebacterineae*, which encompasses *Corynebacterium*, *Mycolicibacterium*, and Mycobacterium.

**FIG 2:**
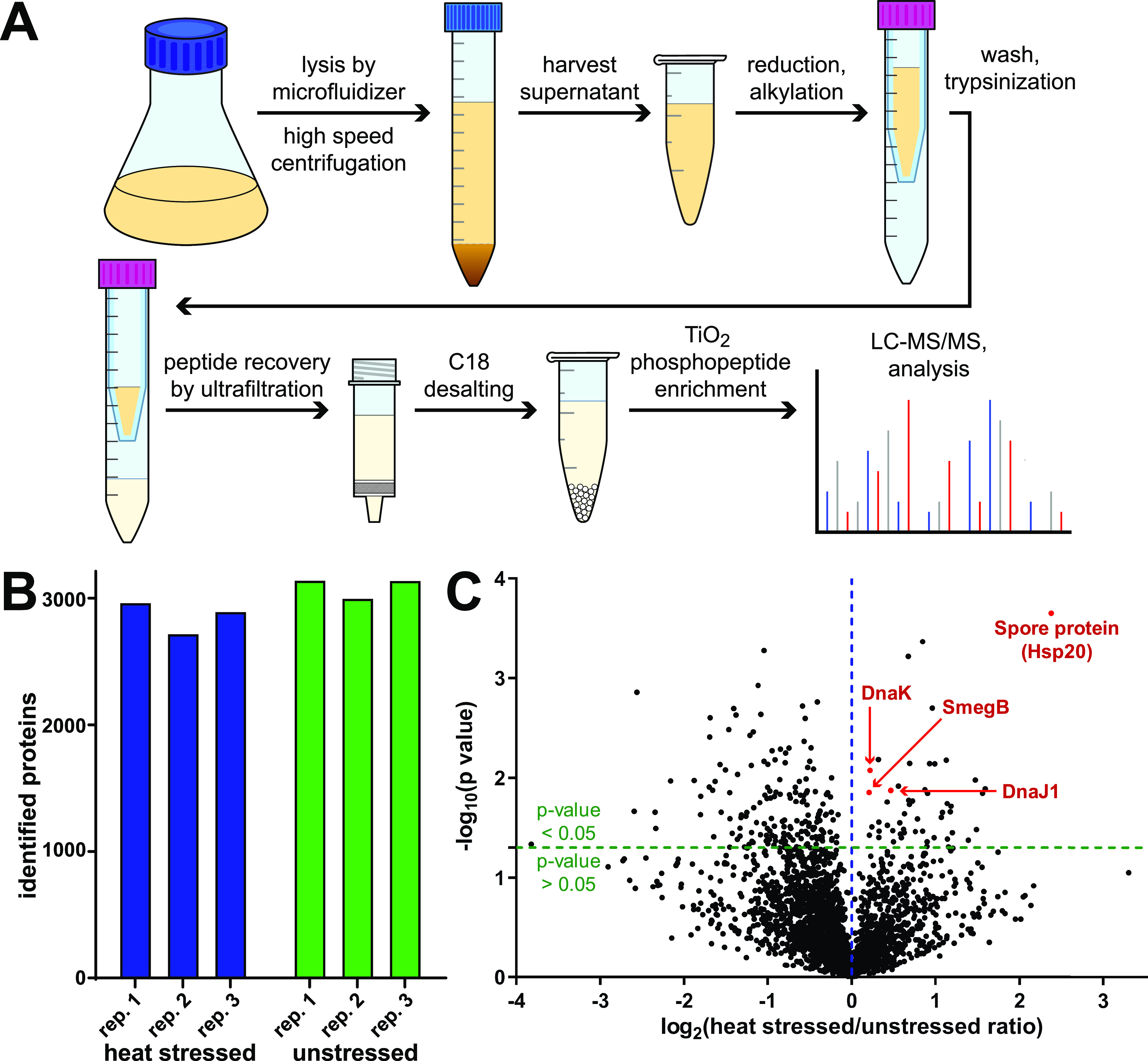
Comparative analysis of mass spectrometry output. (A) Workflow for arginine phosphoproteomics in *Mycolicibacterium smegmatis*. (B) Number of proteins obtained across biological replicates from cells grown under heat stress (50°C) and normal (37°C) conditions. (C) Volcano plot showing enrichment of proteins observed by LC-MS/MS in heat stress versus normal growth conditions. Plotted on the *x* axis is the log_2_ of the ratio of average Sequest HT score of stressed to unstressed samples. The *y* axis shows −log_10_ of *P* values obtained by Student’s *t* test. The horizontal green line indicates a cutoff *P* value = 0.05; the vertical blue line indicates score ratio of 1. Highlighted in red are notable chaperone proteins with stressed/unstressed ratio of >1.

To determine which specific cellular proteins carry pArg modifications in M. smegmatis, we employed an unbiased shotgun proteomics approach. Phosphoarginine is acid labile and has a short half-life in the acidic trifluoroacetic acid (TFA)-containing solvent systems typically used for proteomic sample preparation and liquid chromatography-tandem mass spectrometry (LC-MS/MS) (36). Optimized protocols for arginine phosphoproteomics have been reported that utilize alternative solvent systems at pHs of ≥4 for most steps (34, 36, 39). We adapted these methods to our workflow for sample preparation, phosphopeptide enrichment, and LC-MS/MS, thereby minimizing phosphoarginine hydrolysis (Fig. 2A). Since stress conditions such as heat shock upregulated the occurrence of this modification in B. subtilis (36), we analyzed lysates from M. smegmatis cultures grown either at normal growth temperature (37°C) or under heat stress. In an initial test of M. smegmatis heat tolerance (see Fig. S1 in the supplemental material), we found that cultures grown at 50°C saturated at a lower density but retained substantial viability up to ~20 h. Thus, to increase the likelihood of a robust heat shock response, heat-stressed cultures grown at 50°C for 21 h.

A similar number of proteins (~3,000) were observed in samples from normal growth (3,147, 3,004, and 3,145 proteins in the three replicates) and heat stress conditions (2,969, 2,724, and 2,899 proteins) (Fig. 2B). This suggests that at this level of stress, compensatory stress responses do not involve a complete shutdown of translational machinery or dramatic upregulation of proteolytic activity. Typically, a major part of the stress response to heat shock is the increased expression of heat shock proteins or chaperones. We evaluated the differential levels of observed proteins based on Sequest HT scores. As shown in Fig. 2C, several proteins were observed at significantly higher levels (*P* value ≤ 0.05) in heat shock than under normal growth conditions. These included spore protein (Msmeg_5611), which belongs to the Hsp20 small heat shock protein family and was enriched over 2-fold upon heat shock. Other chaperone proteins had slightly higher scores in heat-stressed samples than in normal growth, including DnaJ1, DnaK, and the SecB-like chaperone SmegB. We also performed comparative Gene Ontology (GO) annotation analysis of strongly enriched/depleted proteins, whose levels differed significantly (ratio > 2; *P* value ≤ 0.05) between conditions (Fig. S2; Table S1). Proteins involved in amino acid, lipid, and noncanonical metabolism, along with redox proteins, were strongly depleted during heat stress. Spore protein was the only stress response protein strongly enriched under heat shock; no protein involved in the stress response was strongly depleted. This differential expression suggests the activation of canonical heat shock response in these samples.

**FIG 3:**
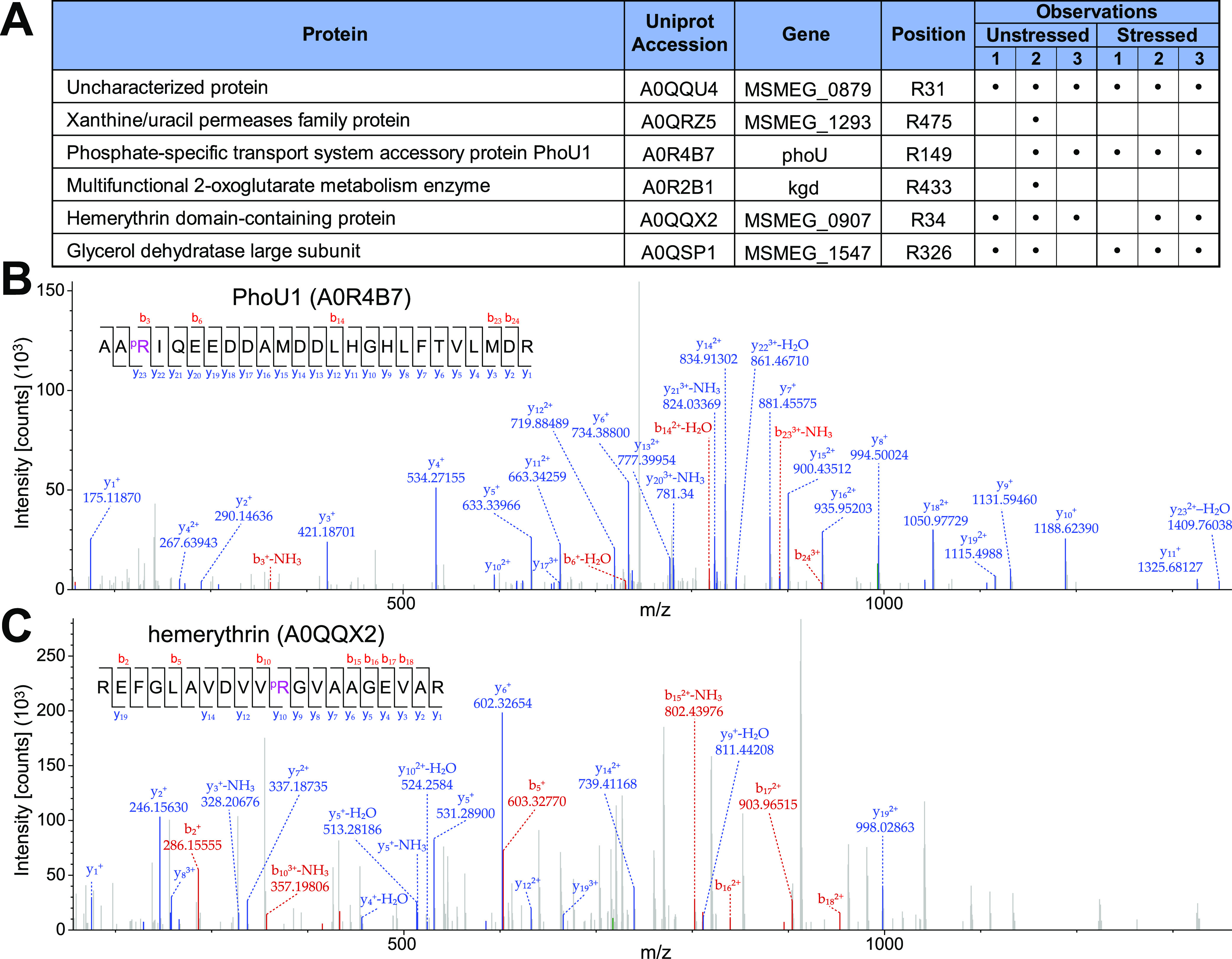
Identification of arginine phosphosites in M. smegmatis proteome. (A) Localized arginine phosphosites in six M. smegmatis proteins. Localization probability is reported as PhosphoRS/ptmRS score. (B and C) Representative secondary fragmentation spectra show arginine-phosphorylated peptides from PhoU1 (B) and hemerythrin domain-containing protein (C). Additional spectra are shown in Fig. S2.

Using Proteome Discoverer, we identified arginine-phosphorylated sites in proteins from these samples. Only peptides with a phosphosite localization probability (PhosphoRS/ptmRS score) of ≥75% were selected (51). We unambiguously localized six phosphoarginine sites in six different proteins (Fig. 3A; Table S2; Fig. S3). Most pArg sites were observed in multiple replicates, in both heat-stressed and unstressed samples (Fig. 3A). The existence of phosphoarginine modifications in our samples implies the existence of an unidentified M. smegmatis pArg kinase. Surprisingly, most pArg sites were observed in both stressed and unstressed samples (Fig. 3A), suggesting that at least these heat stress conditions do not upregulate arginine phosphorylation, in contrast to the paradigm observed in B. subtilis (36).

### Functional characterization of arginine-phosphorylated proteins.

We examined the known or predicted functions of the six proteins observed to carry pArg modifications and found that they have diverse functions, with no obvious common physiological role (Fig. 3A). PhoU1 is a central regulator of the SenX3-RegX3 two-component system responsible for uptake of inorganic phosphate (P_i_) during phosphate starvation (52). This raises the possibility that pArg modifications are linked to P_i_ availability. Two other pArg-bearing targets were metabolic enzymes: the multifunctional 2-oxoglutarate metabolism enzyme Kgd, involved in the tricarboxylic acid cycle (53), and the glycerol dehydratase large subunit (Msmeg_1547), which potentially contributes to the catabolic pathway involving the glycerol dehydration reaction, which yields 3-hydroxypropanal in the presence of adenosylcobalamin coenzyme (Fig. 3A; Fig. S3) (54, 55). pArg may thus play a role in regulating cellular metabolic pathways. Another phosphosite was detected in the transmembrane xanthine/uracil permease (Msmeg_1293), a nucleobase transporter (Fig. 3A and C). A site was found on the hemerythrin domain-containing protein Msmeg_0907, which belongs to a class of O_2_-binding proteins involved in signal transduction, response to H_2_O_2_, oxygen sensing, and nitric oxide reduction (56–58). Finally, a site was observed on Msmeg_0879, a small (48 aa) uncharacterized protein predicted to be predominantly disordered (Fig. 3A and B). Homologs of Msmeg_0879 were detected in close relatives of M. smegmatis but are not widely conserved in actinobacteria.

### Structural and physicochemical analysis of arginine phosphorylation sites.

**FIG 4:**
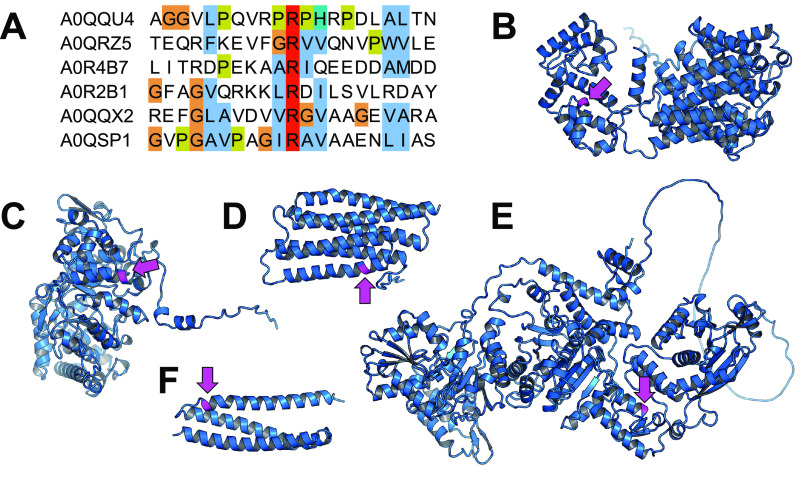
Structural characteristics of phosphoarginine sites. (A) Sequence alignment of 20 residues flanking the arginine phosphosites. (B to F) Structural models of Msmeg_1293 (B), Msmeg_1547 (C), PhoU1 (D), Kgd (E), and Msmeg_0907 (F) are shown with the phosphorylated arginine colored magenta and marked with an arrow. All models were generated by AlphaFold2 (59).

We assessed whether arginine phosphorylation occurred at sites with particular physicochemical properties. We aligned 21-residue sequence segments centered on each unique phosphoarginine site (Fig. 4A) but observe no clear consensus motif. Ala, Leu, Val, and Gly appear to be common in flanking positions, although we note that these are the four most abundant amino acids in the M. smegmatis proteome (13%, 10%, 9%, and 9% of total, respectively) (Table S3). The sequence diversity surrounding pArg positions suggests that target discrimination is guided by characteristics other than primary sequence.

We next examined structural characteristics of phosphorylated positions, based on predicted structures generated by AlphaFold2 (59), except for the small protein Msmeg_0879, for which structure prediction failed. (Notably, the AlphaFold2 prediction for Kgd was virtually identical to its reported X-ray crystal structure [PDB code 2XT6], with a root mean square deviation [RMSD] of ~0.5 Å in the region of the phosphosite [53].) As expected for this charged residue, arginine phosphosites were solvent exposed (Fig. S4) but were located on structured elements rather than loops. Interestingly, in all structures, arginine phosphosites occurred proximal to the beginning or the end of an alpha helix (Fig. 4B to F), which may reflect a structural constraint important for recognition by an arginine kinase.

**FIG 5:**
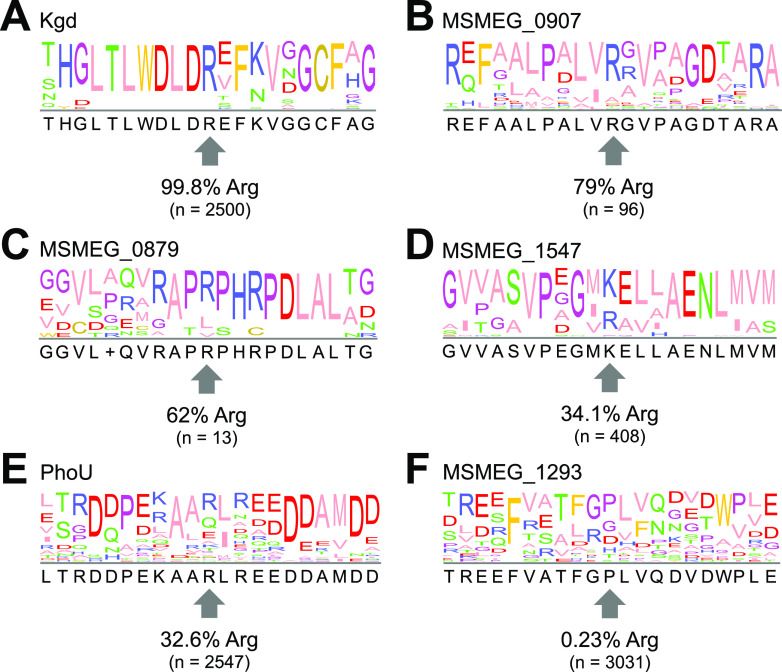
Sequence conservation of phosphorylated arginine residues. Sequence logos show conservation near phosphorylated arginines in Kgd R433 (A), Msmeg_0907 R34 (B), Msmeg_0879 R31 (C), Msmeg_1547 R326 (D), PhoU1 R149 (E), and Msmeg_1293 R475 (F). Percentages indicate positional conservation of the phosphorylated arginine; “n” indicates the number of aligned orthologous sequences used to generate the sequence logo.

Finally, we assessed the sequence conservation of arginine at these positions. We aligned the arginine-phosphorylated M. smegmatis proteins with homologs within the *Corynebacterineae* suborder and analyzed the positional conservation of arginine at the respective positions. As shown in Fig. 5, conservation varies. The phosphosite arginine was well conserved in Kgd (99.8%), Msmeg_0907 (79%), and Msmeg_0879 (62%) (Fig. 5A to C). Conservation was lower in Msmeg_1547 (34.1%) and PhoU1 (32.63%) (Fig. 5D and E). Arg was rarely present in this position in homologs of Msmeg_1293 (0.23%) (Fig. 5F). In cases where the phosphorylated arginine was well conserved, it may indicate that phosphorylation at this position is a conserved phenomenon with functional or regulatory significance.

## DISCUSSION

Protein phosphorylation is a ubiquitous mechanism of signal propagation and pathway regulation in bacteria (60). While examples of His, Asp, Ser, Thr, and Tyr phosphorylation are widespread (61–64), the importance of arginine phosphorylation has become apparent only recently. A growing body of data links pArg modifications to protein quality control pathways and spore germination in *Firmicutes*, including B. subtilis (34, 36–38, 41, 42, 48, 65). However, it has remained unclear how prevalent pArg modifications are in other bacterial phyla. Here, we provide direct evidence for the existence of arginine phosphorylation in *Mycolicibacterium smegmatis*, an actinobacterium.

In B. subtilis and Staphylococcus aureus, this modification is upregulated by stress (34, 36–38, 41, 42, 48, 65). Hence, we performed our study by examining pArg levels in cells grown under both normal conditions and long-term heat stress. While we saw expected increases in levels of some heat shock proteins overall, arginine phosphorylation abundance was not altered by heat stress, as occurs in *Firmicutes*. It is possible that pArg modifications play no role in the mycobacterial stress response and instead occur in the context of targeted pathway regulation. On the other hand, we cannot rule out a role for pArg in other stress contexts that were not tested in this study, including milder heat shock conditions. Notably, the sustained heat stress conditions in this study may have impaired the activity of the yet-unidentified mycobacterial arginine kinase.

The six arginine phosphosites localized in this work were far fewer than those identified in other bacterial studies. Two independent studies in B. subtilis observed 121 and 217 sites in 87 and 134 proteins, respectively (36, 41), while in S. aureus 207 sites were identified in 126 proteins (37). On its face, the small number of pArg modifications found in M. smegmatis argues against a proteome-wide quality control role and appears more consistent with targeted regulation of selected proteins and processes. However, we note that phosphoproteomic studies with *Firmicutes* utilized deletion strains lacking known arginine phosphatases (B. subtilis YwlE and S. aureus PtpB), which elevate levels of pArg-bearing proteins. No arginine phosphatase has yet been identified in M. smegmatis or M. tuberculosis; thus, our approach utilized wild-type M. smegmatis. If a mycobacterial arginine phosphatase is eventually identified, it would be interesting to observe the effect of its knockout or knockdown on pArg levels.

For arginine phosphorylation to be useful to the cell, it must presumably be applied selectively through the regulated activity of arginine kinases. Many protein kinases recognize substrates through characteristic sequence motifs surrounding the phosphosite. In contrast, our examination of mycobacterial arginine phosphosites revealed no clearly enriched consensus sequence around pArg. Prior analysis of Arg phosphosites in B. subtilis similarly revealed no evident consensus sequence (36). This degeneracy suggests either that distal interactions guide M. smegmatis arginine kinases to phosphosites or that other characteristics of target proteins guide substrate selection. Supporting the latter possibility, we noted that all phosphorylated arginine residues (for which structural models could be obtained) were found near one end of an alpha helix, which may indicate a structural feature recognized by an arginine kinase.

What role does arginine phosphorylation play in mycobacterial cells? In B. subtilis, pArg tagging helps enforce protein quality control by marking misfolded proteins for destruction by ClpCP (34, 36, 41). Prior studies have demonstrated that mycobacterial ClpC1P1P2 can degrade model substrates *in vitro* bearing pArg modifications (42). The pArg-bearing proteins identified in this work may therefore be recognized as proteolytic substrates by ClpC1P1P2. Future studies will be required to test whether the pArg-bearing population of these proteins experiences increased ClpC1-dependent turnover. Alternatively, pArg may modulate some aspect of the targets’ function or interactions with other proteins. We observed pArg on PhoU1, which helps regulate P_i_ uptake by inhibiting the activity of the SenX3-RegX3 two-component system when P_i_ is readily available (52). Additionally, the signal transduction protein Msmeg_0907, metabolic proteins (Kgd and Msmeg_1547), a transmembrane xanthine/uracil transport protein (Msmeg_1293), and an uncharacterized protein (Msmeg_0879) were found to be phosphorylated. In terms of essentiality, a prior study showed that the M. tuberculosis homolog of Kgd is essential for viability (2); another report showed that PhoU1 is jointly essential with the PhoU2 protein (Msmeg_1605) for *in vitro* M. smegmatis growth (52). More work is required to determine whether pArg modifications regulate these pathways and whether such modification plays an essential role in mycobacterial physiology. Along other lines, recent studies point to a role for the ClpC1 NTD in regulating unfoldase activity by modulating the formation of higher-order ClpC1 oligomers (66). Binding of pArg to the NTD may thus influence the overall activity state of the enzyme toward other targets.

This study lays the groundwork for future efforts to expound on the roles of phosphoarginine modifications in mycobacteria. The immediate impediment to further understanding this system is the absence of identifiable orthologs of known arginine kinases or phosphatases. Nevertheless, the lack of consensus motif in the identified phosphosites predicts a promiscuous kinase that targets cellular proteins, in a manner independent of a specific primary sequence. Future work will be required to decipher how arginine phosphorylation is regulated and how it contributes to the physiology of mycobacteria and other actinobacterial species.

## MATERIALS AND METHODS

### ClpC1 sequence analysis.

Actinobacterial homologs of M. smegmatis ClpC1 were identified using HMMER (67) and aligned using Clustal Omega (68). Fragmentary sequences were omitted from analysis. To reduce overrepresentation of similar taxa (e.g., multiple M. tuberculosis strains), alignments were pruned such that no two sequences had greater than 90% sequence identity. Positional conservation scores were exported from Jalview (69) and plotted as a heatmap in Prism (GraphPad). The sequence logo of the NTD region was generated using WebLogo (70).

### Protein purification and binding assays.

The nucleotide sequence encoding the NTD of ClpC1 (codons 1 to 147; referred to as ClpC1^NTD^) was amplified from M. smegmatis MC^2^155 genomic DNA (gDNA) (ATCC) and cloned into a pET22b-derived vector (EMD Millipore) in frame with a C-terminal LPETGG sortase recognition sequence (71) and 6×His tag. ClpC1^NTD^ was overexpressed in E. coli strain ER2566 (New England BioLabs [NEB]) by induction with 0.5 mM isopropyl β-d-1-thiogalactopyranoside at 30°C for 4 h. Cells were harvested by centrifugation, resuspended in lysis buffer (25 mM HEPES, 500 mM NaCl, 10 mM imidazole, 10% glycerol [pH 7.5]), and lysed by sonication. ClpC1^NTD^ was purified from clarified lysate by nickel-nitrilotriacetic acid (Ni-NTA; Marvelgent Biosciences) and anion exchange (Source Q; Cytiva) chromatography. ClpC1^NTD^ was fluorescently labeled via sortase transpeptidation (71) by 2 h of incubation with sortase A (~1:30 molar ratio) and a 2-fold molar excess of a Gly-Gly-Asn-Lys-(fluorescein isothiocyanate) peptide (Biomatik) in PBS buffer (10 mM Na_2_HPO_4_, 1.8 mM KH_2_PO_4_, 137 mM NaCl, 2.7 mM KCl, 10% glycerol [pH 7.4]). Excess peptide was removed by size exclusion chromatography (Superdex 75; Cytiva). Purified ClpC1^NTD-FITC^ was concentrated and stored in CPD buffer (25 mM HEPES, 200 mM NaCl, 10 mM MgCl_2_, 0.1 mM EDTA [pH 7.0]). Binding of phosphoarginine (Millipore Sigma) to 0.1 μM purified ClpC1^NTD-FITC^ was assayed in CPD buffer supplemented with 0.05% Tween 20 and 8 mg/mL of bovine serum albumin (BSA) by microscale thermophoresis using a Monolith NT.115 (NanoTemper) (72). Thermophoretic data were fit to a quadratic single site binding equation in Prism (GraphPad).

### Cell culture conditions.

Liquid starter cultures of M. smegmatis MC^2^155 were prepared in 20 mL of Middlebrook broth base (HiMedia) containing 0.2% (vol/vol) glycerol (Fisher Scientific) and 0.05% (vol/vol) Tween 80 and grown for 48 h at 37°C with orbital shaking at 250 rpm. Saturated starter cultures were subcultured into 900 mL of fresh medium at a starting *A*_600_ of ~0.02 and grown at 37°C until reaching an *A*_600_ of ~1.0. A total of 500 mL of culture was collected and added to 400 mL of fresh medium. Unstressed control samples were further grown at 37°C, while samples for heat stress were grown at 50°C for 21 h. Cells were harvested by centrifugation at 9,000 × *g* for 20 min at 4°C, and pellets were resuspended in 5 mL of ice-cold lysis buffer (25 mM HEPES, 200 mM KCl, 10 mM MgCl_2_, 0.1 mM EDTA [pH 7.5]) containing 10 mM ATP (Fisher), 200 μL of phosphatase inhibitor cocktail (Millipore Sigma), 30 mM sodium pervanadate (Acros Organics), 3 mM NaF, and 3 mM sodium pyrophosphate (both from Fisher). Cells were lysed at high pressure using a microfluidizer (Microfluidics). Lysates were clarified at 15,000 × *g* for 30 min at 4°C, and supernatant was stored at −80°C prior to further processing. Total protein content was estimated by Bradford assay (Bio-Rad).

### Filter-aided sample preparation for mass spectrometry.

Replicate samples of clarified cell lysates (approximately 10 mg total protein) were prepared for mass spectrometry using filter-aided sample preparation (FASP) (36, 73). Reduction of disulfide bonds was achieved by the addition of 200 mM dithiothreitol (DTT; Fisher), followed by incubation at 56°C for 50 min. Afterwards, samples were diluted in 7 mL of 0.1 M Tris (pH 7.5; Gold Biotechnology) containing 8 M urea and 25 mM 2-iodoacetamide (both from Acros Organics) in 15-mL tubes and incubated in the dark at room temperature for 45 min to carbamidomethylate cysteines. After alkylation, samples were transferred to an Amicon filter (10,000-molecular-weight cutoff [MWCO]; Millipore Sigma), washed twice with 5 mL of 0.1 M Tris (pH 7.5) containing 8 M urea by centrifugation at 4,000 × *g*, then washed twice with 5 mL of 0.1 M Tris (pH 7.5) containing 4 M urea, and finally washed twice with 5 mL of 100 mM ammonium bicarbonate (Honeywell). After the last wash step, retentate was reduced to less than 1 mL by spin concentration.

### In-solution trypsin proteolytic cleavage.

MS-grade trypsin protease (Pierce) was dissolved to obtain a 20-μg/mL stock in 100 mM ammonium bicarbonate. Trypsin digestion of processed M. smegmatis lysates was performed in an Amicon filter unit at a protein/trypsin ratio of approximately 1,500:1 overnight at 37°C. Upon digestion, peptides were recovered by centrifugation at 4,000 × *g* for 10 min, followed by the addition of 0.5 M NaCl. Eluted samples were dried under vacuum in a SpeedVac centrifuge (Thermo Fisher).

### TiO_2_ enrichment of phosphopeptides.

Phosphopeptides were enriched from tryptic peptide samples using Titansphere TiO_2_ beads (5 μm; GL Sciences) under buffer conditions that limited acid hydrolysis of phosphoarginine (34, 36, 74). Five milligrams of TiO_2_ resin was resuspended in 1 mL of binding buffer (300 mg/mL lactic acid, 12.5% acetic acid, 60% acetonitrile, 0.2% heptafluorobutyric acid [pH 4] with NH_4_OH). Lyophilized peptide samples were redissolved in the TiO_2_ suspension, incubated for 35 min at 20°C with gentle agitation, and then transferred to graphite spin columns (Thermo Fisher). Unbound peptides were removed by a wash step with 150 μL of binding buffer and spun at 2,000 × *g* for 1 min, followed by three wash steps using 400 μL of wash solution A (200 mg/mL of lactic acid, 75% acetonitrile, 2% trifluoroacetic acid, 2% heptafluorobutyric acid), wash solution B (200 mg/mL of lactic acid, 75% acetonitrile, 10% acetic acid, 0.1% heptafluorobutyric acid [pH 4] with NH_4_OH), and wash solution C (80% acetonitrile, 10% acetic acid). The resin was then incubated with 100 μL of elution solution 1 (1% NH_4_OH, 30 mM ammonium phosphate) and elution solution 2 (1.25% NH_4_OH in 50% acetonitrile) for 15 min each. The eluate containing phosphopeptides was collected by centrifugation after each incubation. To remove salts, samples were desalted using a HyperSep C_18_ column (Thermo Scientific). Samples were lyophilized and stored at −80°C prior to mass spectrometry.

### LC-MS/MS.

Lyophilized and desalted tryptic digests were resuspended in 20 μL of 0.5% acetic acid (pH 4.5). An Orbitrap Eclipse mass spectrometer (MS; Thermo Scientific) coupled with an Ultimate 3000 nano-liquid chromatography (nano-LC) system and a FAIMS Pro Interface (Thermo Scientific) was used for the LC-tandem mass spectrometry (MS/MS) analysis. Peptide samples were first loaded onto a trap column (PepMap C_18_; 2 cm by 100 μm [inside diameter]) and afterwards separated at a flow rate of 300 nL/min on an analytical column (PepMap C_18_, 3.0 μm; 10 cm by 75 mm [inside diameter]; Thermo Scientific). A binary buffer system (buffer A, 0.1% formic acid in water; buffer B, 0.1% formic acid in acetonitrile) with a 165-min gradient (1% to 25% buffer B over 125 min, 25% to 32% buffer B in 10 min, then 95% buffer B over 3 min, back to 1% B in 5 min, and stay equilibration at 1% buffer B for 20 min) was utilized. To achieve field asymmetric ion mobility spectrometry (FAIMS) separation, multiple compensation voltages [CVs] (−45, −60, and −80) were applied. For all experiments, the survey scans (MS1) were acquired over a mass range of 375 to 1,500 *m/z* at a resolution of 60,000 in the Orbitrap. Isolation of precursors was done with a width of 1.6 *m/z* for MS/MS acquisition. The precursors were subsequently fragmented with higher-energy collisional dissociation (HCD) using 30% collision energy with a maximum injection time of 100 ms and collected in Orbitrap at 15,000 resolution. The dynamic exclusion was set to 60 s and was shared across different FAIMS experiments. LS-MS/MS data were collected in independent biological triplicates.

### Mass spectrometry data analysis.

Proteomic analysis was performed in the Proteome Discoverer software suite (version 2.2; Thermo Fisher). Raw data were searched against the M. smegmatis (strain ATCC 700084 [MC^2^155]) UniProt Reference Proteome (Proteome identifier UP000000757; 6,602 entries in total) using Sequest HT (University of Washington and Thermo Fisher) (75). Iodoacetamide-mediated cysteine carbamidomethylation was set as a static modification, while methionine oxidation and phosphorylation of arginine, serine, threonine, tyrosine, and histidine residues were entered as dynamic modifications. Complete trypsinization with a maximum of two missed cleavages was allowed. Precursor mass tolerance was set at 10 ppm while allowing fragment ions to have a mass deviation of 0.02 Da for the HCD data. Validation of peptide-spectrum matches (PSM) based on *q* value was done using Percolator, with target false-discovery rates (FDR) of 1% and 5% for stringent and relaxed validations, respectively. The false-discovery rate of high-confidence protein and peptide identification was 1%. Localization probability of phosphopeptide hits was analyzed using the PhosphoRS (ptmRS) node of the Proteome Discoverer software (76). Only modifications with a PhosphoRS (ptmRS) score of ≥75% were selected.

### Gene Ontology annotation analysis.

Gene Ontology (GO) annotation was performed using the OmicsBox software suite (77). Homologs were identified by BLAST (Basic Local Alignment Search Tool) search against the Swiss-Prot/UniProt database (78), with a minimum expectation value 10^−3^. Homolog annotations were compiled from the InterPro database (79). BLAST and InterPro results were used to generate GO terms in terms of biological process. For proteins still unannotated, direct UniProt BLAST was performed, and tentative assignment of functional group was based on those of obtained homologs.

### Sequence and structural analysis of arginine phosphosites.

For alignment analysis, a 21-mer sequence was obtained containing 10 residues flanking each side of phosphoarginine. All 21-mers were then aligned in BioEdit (80). Structural information, if available, was obtained from Protein Data Bank (PDB) (81). Predicted structures were retrieved using a neural network-based machine learning model, AlphaFold (version 2 [59]), and molecular images were prepared in PyMOL (version 2.5.2; Schrödinger). Protein BLAST of arginine-phosphorylated proteins was performed in the NCBI suite (82). Analysis was restricted to the *Corynebacterineae* suborder. Sequences were aligned using Clustal Omega (68), and conservation and consensus values were obtained in Jalview (69).

### Data availability.

The mass spectrometry data from this publication have been submitted to the ProteomeXchange Consortium (http://proteomecentral.proteomexchange.org) via the PRIDE partner repository (83) and assigned the identifier PXD032083.
