# Supplementary material for: Identification of Arginine Phosphorylation in Mycolicibacterium smegmatis
Source: Microbiol Spectr. 2022 Oct 10;10(5):e02042-22. doi: 10.1128/spectrum.02042-22 (PMC9604228; doi:10.1128/spectrum.02042-22)
Supplement: Supplemental Material — Fig. S1 to S4. Download spectrum.02042-22-s0005.pdf, PDF file, 4.3 MB [file spectrum.02042-22-s0005.pdf]

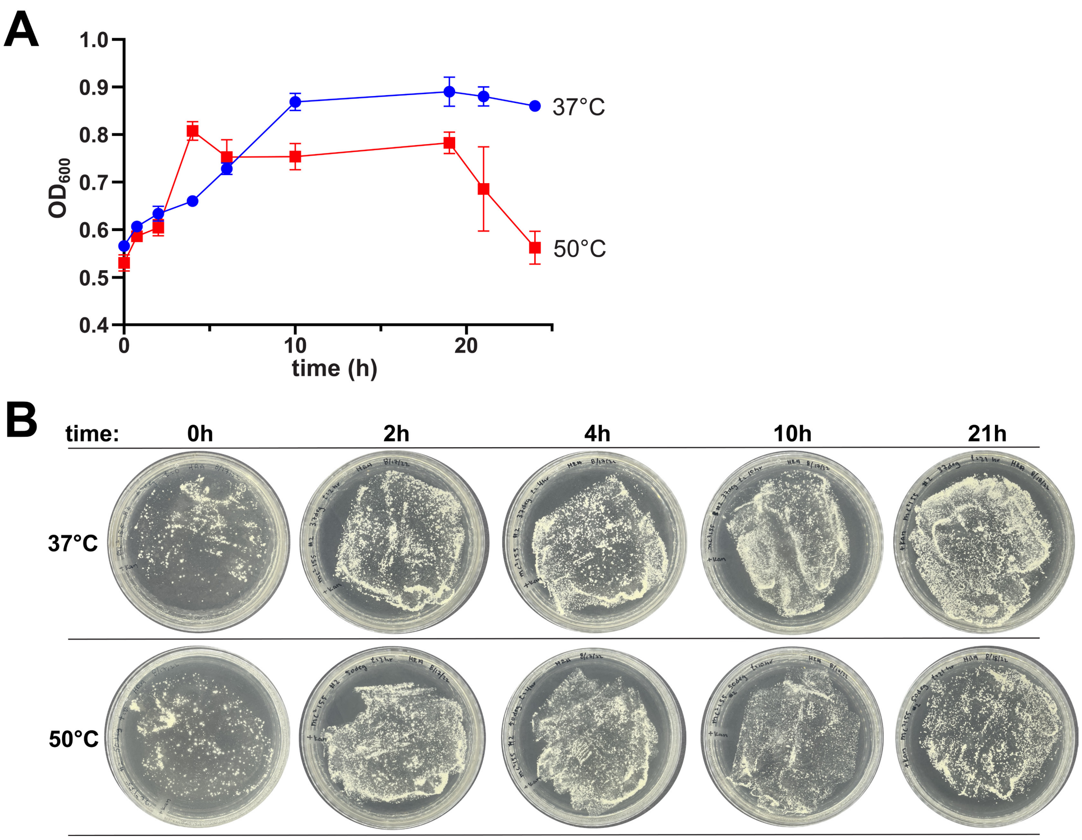

Supplemental Figure S1. *M. smegmatis* cultures retain viability during long term heat stress. Liquid cultures of *Msm* were grown at 37C or 50C for 24 h. (**A**) OD_{600} measurements were taken over the course of growth and (**B**) $3x10^{-6}$ OD•mL samples were plated to determine viability. Notably, clumping occurred in the heat stressed culture around 20 h, which introduces uncertainty into the OD_{600} measurements and prevents quantitative assessment of colony forming units.

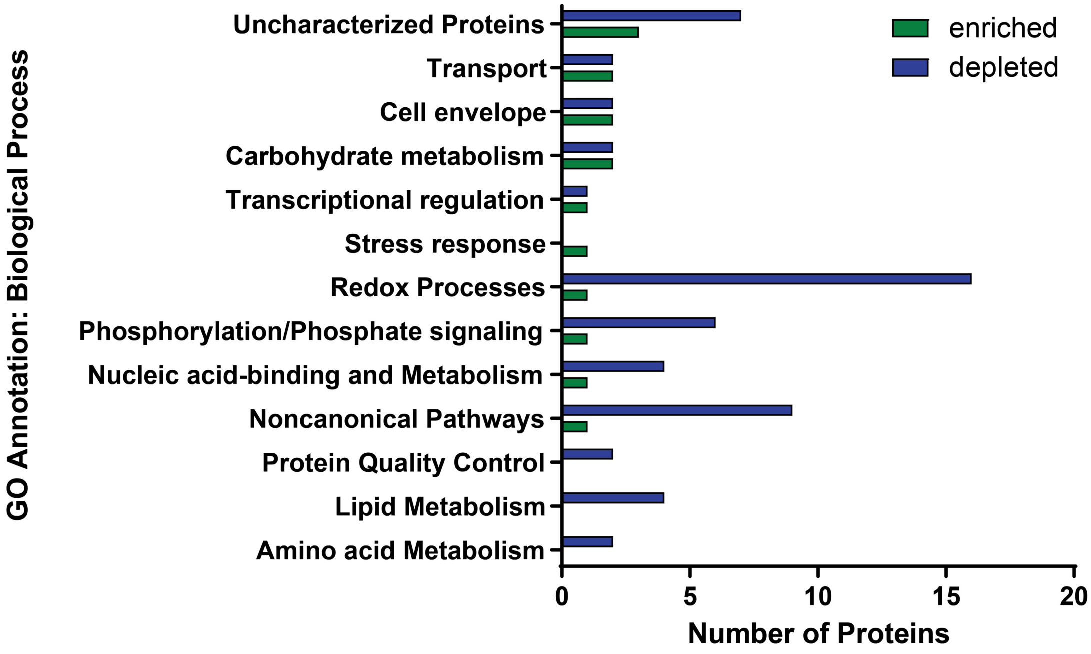

Supplemental Figure S2. Gene ontology (GO) annotation of proteins strongly enriched or depleted upon heat shock. Proteins with significantly ($p \leq 0.05$) and strongly different levels (ratio > 2) in heat shock and control conditions were sorted into GO annotation categories mostly based on biological process.

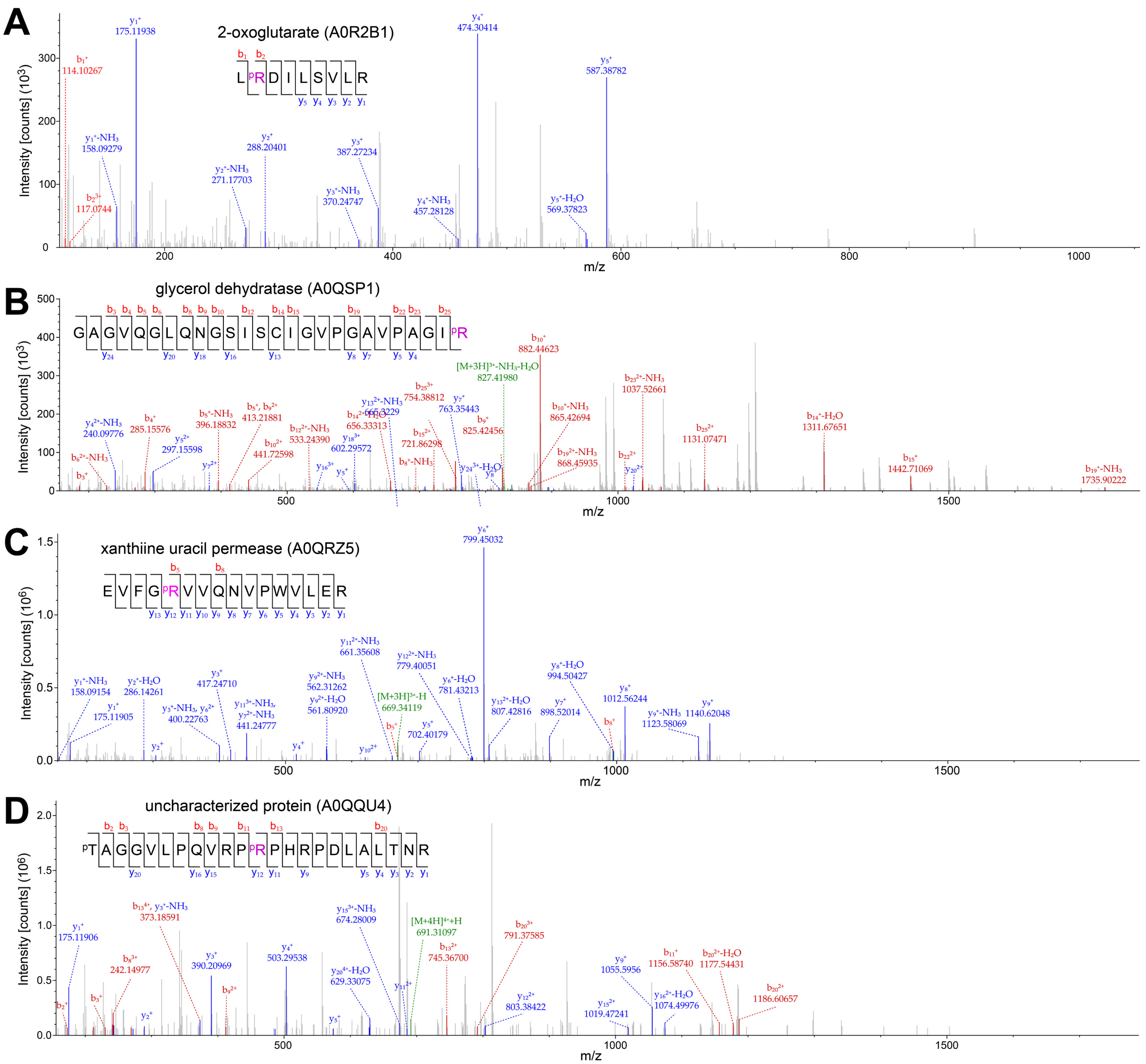

Supplemental Figure S3. Phosphosite secondary spectra. Secondary fragmentation spectra showing arginine-phosphorylated peptides in (**A**) 2-oxoglutarate, (**B**) glycerol dehydratase, (**C**) xanthine uracyl permease, and (**D**) MSMEG_0879.

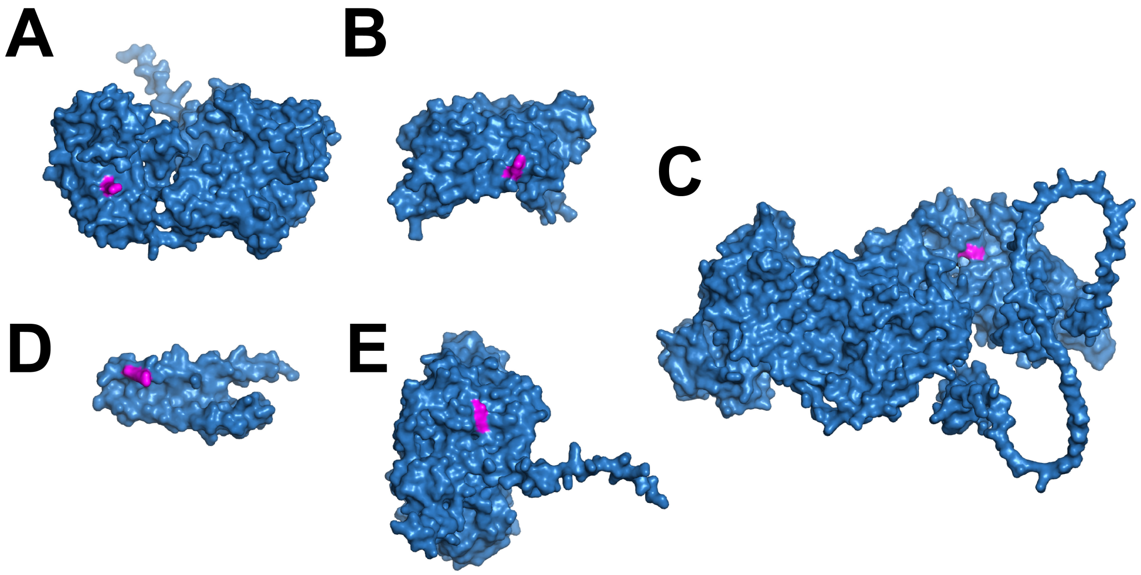

Supplemental Figure S4. Solvent accessibility of phosphorylated arginines. Surface representations of proteins (**A**) MSMEG_1293 (**B**) PhoU1 (**C**) Kgd (**D**) MSMEG_0907 (**E**) MSMEG_1547 based on AlphaFold2 prediction. Magenta patches represent specific arginine residues observed to be arginine-phosphorylated located on protein surface.
